# Molecular regulators of thromboinflammation and angiogenesis in pediatric cancer: emerging roles of noncoding RNAs, epigenetics, and extracellular vesicles – narrative review

**DOI:** 10.1097/MS9.0000000000004853

**Published:** 2026-04-20

**Authors:** Shreya Singh Beniwal, Prashasti Dahiya, Manju Ramakrishnan, Rafael Everton Assunção Ribeiro da Costa, Akash Rawat, Yujin Jeong, Rajya Lakshmi Devarapalli, Alexandros Keramidas, Chimuka Mwaanga, Aarushi Mishra

**Affiliations:** a Lady Hardinge Medical College, New Delhi, India; b ESIC Medical College and Hospital, Faridabad, Haryana, India; c Emory University, Atlanta, Georgia, USA; d State University of Campinas, Campinas, São Paulo, Brazil; e Cidade Universitária “Zeferino Vaz”, Campinas, São Paulo, Brazil; f Himalayan Institute of Medical Sciences, Swami Rama Himalayan University, Dehradun, India; g Icahn School of Medicine at Mount Sinai/Elmhurst Hospital Center, New York City, New York, USA; h Andhra Medical College, Visakhapatnam, Andhra Pradesh, India; i Aristotle University of Thessaloniki, Thessaloniki, Greece; j Texila American University Zambia, Lusaka, Zambia; k Lvivs’kyj Nacionaľnyj Medychnyj Universytet imeni Danyla Halyc’koho, Lviv, Ukraine

**Keywords:** angiogenesis, extracellular vesicles, noncoding RNAs, pediatric cancer, thromboinflammation

## Abstract

Pediatric cancers, although less prevalent, remain a significant cause of mortality among children worldwide. Thromboinflammation arises from a complex dysregulation of inflammation and hemostasis, leading to tissue injury and cell death. Angiogenesis, the formation of new blood vessels, is influenced by inflammation. Both processes are closely linked to the progression of various tumors. Emerging evidence increasingly highlights the pivotal role of certain molecular markers in stimulating thromboinflammatory and angiogenic pathways in several types of pediatric cancers, including gliomas, other brain tumors, retinoblastoma, leukemia, lymphoma, and other solid malignancies. Current studies show that noncoding RNAs (ncRNAs), extracellular vesicles (EVs), and epigenetic dysregulation of key biological pathways in specific genes contribute to alterations in the tumor microenvironment. These changes are crucial for tumor initiation, progression, and dissemination through immune-mediated mechanisms that promote thromboinflammation and angiogenesis. The detailed identification and assessment of the expression profiles of these markers hold growing potential to support the diagnosis of various pediatric cancers, provide better prognostic stratification, and guide targeted therapeutic approaches. Therefore, integrating this knowledge into existing strategies for diagnosis, staging, and treatment – especially through liquid biopsy based on EVs and RNA signatures – may enable earlier and more precise interventions. This has the potential to positively impact long-term survival and quality of life.

## Introduction

Although pediatric cancers are less prevalent than adult malignancies, they continue to be a major cause of cancer-related deaths in children worldwide. Unlike adult tumors, which are often associated with environmental exposures or lifestyle factors, pediatric cancers usually emerge spontaneously due to alterations in developmental and molecular pathways^[^1^]^. While advancements in treatment have improved survival rates, many pediatric cancer survivors face lasting health complications, underscoring the importance of deepening our understanding of the biological mechanisms behind these aggressive diseases.

HIGHLIGHTS

- Pediatric cancers, though less common than adult malignancies, exhibit significant thromboinflammatory and angiogenic activity contributing to tumor progression.
- ncRNAs, EVs, and epigenetic modifications are emerging as key molecular regulators of thromboinflammation and angiogenesis.
- These molecular markers influence tumor microenvironment, immune responses, and vascular remodeling in pediatric malignancies.
- Profiling these markers offers potential for improved diagnosis, prognostic stratification, and development of targeted therapies.
- Integration of EV- and RNA-based liquid biopsy approaches may enable earlier, precise interventions, potentially improving long-term outcomes.

A growing area of interest is thromboinflammation, the interconnected relationship between the body’s coagulation system and inflammatory responses, and its role in promoting tumor progression. In pediatric cancers, tumor cells can activate coagulation through tissue factor (TF) expression or by damaging blood vessels. Inflammatory cytokines then enhance thrombin production, creating a feedback loop that supports tumor growth, metastasis, and immune escape^[^2,3^]^. Children with cancer are also at a markedly higher risk of thrombosis (up to 30 times that of healthy children), yet much of the mechanistic insight comes from studies in adult populations^[^4^]^.

Alongside thromboinflammation, angiogenesis, which is the formation of new blood vessels, largely driven by VEGF signaling, is essential for tumor growth and survival. This is particularly crucial in pediatric cancers such as retinoblastoma and medulloblastoma, which are highly vascularized and rely on a persistent blood supply^[^2^]^. Research increasingly indicates that inflammatory and coagulation pathways contribute to angiogenic processes, hinting at shared molecular regulators orchestrating these intertwined events within the tumor microenvironment (TME).

Recent studies highlight the significance of noncoding RNAs (ncRNAs), epigenetic modifications, and extracellular vesicles (EVs) as key regulators of both thromboinflammatory and angiogenic pathways in cancer^[^5–7^]^. These elements influence immune responses, endothelial behavior, and vascular remodeling. These functions are central to pediatric tumor development. Their stability and functional specificity make them promising tools for identifying biomarkers, refining risk assessment, and developing targeted therapies.

This review aims to summarize what is currently known about these mechanisms in pediatric cancers and explores how they could help improve diagnosis, treatment planning, and targeted therapies.

## Thromboinflammation and angiogenesis in pediatric cancer

### Thromboinflammation and angiogenesis:

Thromboinflammation can be defined as the complex crosstalk between thrombosis, inflammation, and vascular permeability, wherein each process amplifies the others, culminating in tissue injury and death. It is a pathological process that arises from the dysregulation of two otherwise tightly regulated and interdependent physiological systems: inflammation and hemostasis^[^8^]^. Angiogenesis, the sprouting of new blood vessels from pre-existing vasculature, is intricately linked to inflammation through immune cell-mediated signaling cascades. Inflammation induces angiogenesis by activating different cell populations that secrete angiogenic factors, leading to neovascularization^[^9,10^]^.

### Cancer-associated coagulopathy and platelet activation

**Figure 1.**
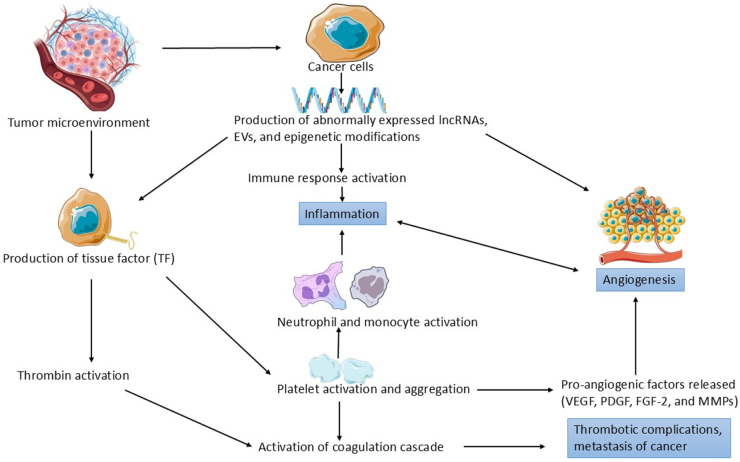
Conceptual diagram illustrating the complex interconnections between thromboinflammation, angiogenesis, and molecular regulators (ncRNAs, EVs, and epigenetics) within the TME.

Cancer cells promote hypercoagulability via secretion of procoagulants, especially TF. Clinical studies have shown that TF is overexpressed on the surface of malignant cells across many cancer types, and this overexpression is positively associated with disease progression^[^11,12^]^. TF binds coagulation factors circulating in the plasma, initiating a cascade that culminates in thrombin generation. Thrombin has a plethora of cellular effects, as it cleaves fibrinogen and leads to the activation of protease-activated receptors (PARs) PAR-1, -3, and -4. These receptors are G protein-coupled, and upon activation, lead to the release of growth factors. Thrombin also activates platelets using these receptors^[^11^]^. Besides, cancer cells have also been known to generate certain procoagulant microparticles that lead to thrombin generation. They also secrete platelet agonists that enhance tumor cell–platelet interactions and increase the adhesive capabilities of both cell types. This leads to the recruitment of even more platelets at the site of interaction, ultimately resulting in platelet aggregation^[^11^]^. Activated platelets secrete inflammatory mediators, coagulation-promoting molecules, and growth factors that contribute to the initiation and amplification of thromboinflammation^[^8^]^. These activated platelets are also responsible for distant metastasis of the tumor via thrombocytosis^[^9^]^. Platelet cloaking of tumor cells facilitates immune evasion and promotes their adhesion to the endothelium, aiding in metastasis^[^13^]^. In the TME, these platelets release certain factors that promote angiogenesis, including platelet-derived growth factor (PDGF), fibroblast growth factor (FGF), and metalloproteinases (MMPs). The alpha and dense granules of platelets serve as reservoirs for potent proangiogenic molecules, including vascular endothelial growth factor (VEGF), PDGF, fibroblast growth factor-2 (FGF-2), and MMPs, which are released upon activation to initiate neovascularization in the TME^[^14^]^. These newly developed blood vessels help sustain inflammation by facilitating the movement of immune cells into the affected area^[^10^]^. These processes are not isolated; coagulation and inflammation synergistically drive angiogenesis, sustaining tumor growth and facilitating metastasis^[^9,13,14^]^. In addition, activated platelets recruit monocytes and granulocytes to sites of cancer cell arrest, collaborating to establish a pro-metastatic microenvironment^[^15,16^]^. Figure 1 highlights the interconnections between thromboinflammation, angiogenesis, and their role in metastasis of cancer.

### Thromboembolism and angiogenesis in pediatric cancers

Thromboembolic events associated with cancer are being reported more frequently in children. Pediatric cancer patients may have as much as a 30-fold higher risk of developing thromboembolism compared to children without malignancy^[^8^]^. The prevalence of cancer-associated thrombosis in pediatric cancer patients has been reported to range from up to 16% in symptomatic cases to as high as 40% in those without symptoms^[^17^]^. In the pediatric population, these thromboembolic events are most commonly observed in hematologic cancers, especially acute lymphoblastic leukemia (ALL) and lymphomas, but they also occur in solid tumors like sarcomas. The location of thromboembolic complications depends on the cancer type. In 50% of the patients with ALL who have thromboembolism, this location is the central nervous system, leading to neurologic complications. It has also been described in 3% of non-Hodgkin’s lymphoma patients^[^1,18^]^. Angiogenesis plays an important role in both metastasis and tumor growth. It is especially important in pediatric brain tumors like pilocytic astrocytoma and high-grade gliomas, which are highly vascularized. Other highly vascularized pediatric brain tumors include neuroblastoma, Wilms’ tumor, soft-tissue sarcomas such as rhabdomyosarcoma, osteosarcoma, and Ewing’s sarcoma. Optimal antiangiogenic therapy is the cornerstone of the treatment of these tumors^[^19^]^.

### Chemotherapy-induced endothelial dysfunction

**Figure 2.**
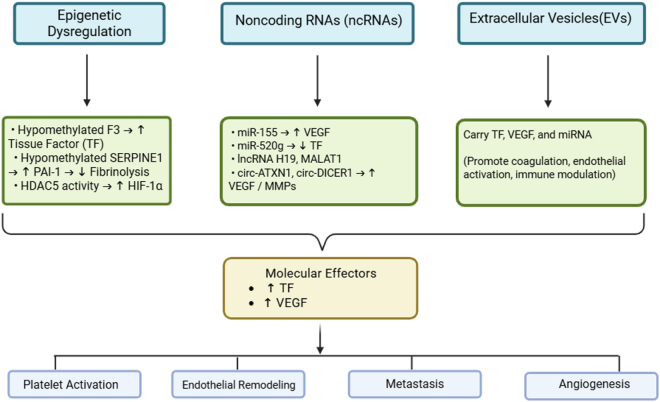
Illustration of upstream epigenetic and RNA-mediated dysregulation converging on thromboinflammatory and angiogenic pathways in pediatric tumors. These include TF and VEGF overexpression, platelet activation, and endothelial remodeling, resulting in tumor progression, immune evasion, and metastasis.

**Figure 3.**
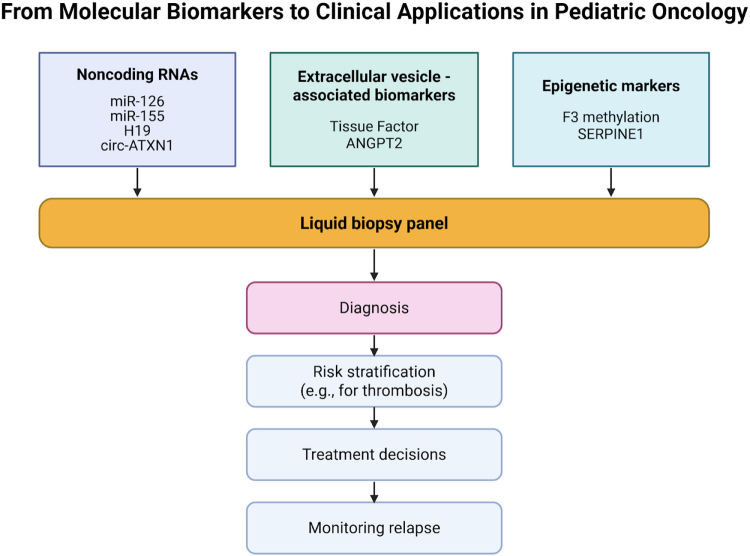
Flow diagram showing the translational pathway from molecular regulators – such as EVs, ncRNAs, and epigenetic markers – to clinical utility in pediatric oncology via liquid biopsy, biomarker panels, and individualized therapy^[^5–7,42,49,68,71,88^]^.

Certain chemotherapeutic agents, such as anthracyclines, have been known to cause endothelial cell (EC) dysfunction and injury in pediatric cancer patients^[^20,21^]^. The long-term consequences of this complication include cardiovascular disorders. It also promotes thrombus formation and inflammation by releasing agents such as plasminogen activator inhibitor 1 (PAI-1), platelet-activated factor 4 (PF-4), and interleukins (IL-1 and IL-6). Thus, chemotherapeutic agents can themselves have procoagulant effects^[^22^]^. This chemotherapy-induced EC activation has also been implied as a cause of angiogenesis (new vessel formation) in some studies, owing to the stimulation of various proangiogenic signaling pathways^[^23^]^. These intricate vascular responses in pediatric cancers underscore the relevance of upstream molecular regulators – such as ncRNAs, epigenetic mechanisms, and EVs – which modulate the thromboinflammatory and angiogenic landscape^[^24–26^]^. Figure 2 illustrates the mechanistic convergence of epigenetic modifications, ncRNAs, and EVs on thromboinflammatory and angiogenic pathways in pediatric cancers. The mechanistic convergence of these regulators is illustrated in Figure 2, while their translation into clinical applications—including liquid biopsy, biomarker development, and individualized therapy—is depicted in Figure 3.

## Role of ncRNAs

ncRNAs affect the growth and spread of tumor cells via various mechanisms. MicroRNAs have been found to regulate the coagulation cascade. For example, in a study, miR-520g was found to reduce the expression of TF and procoagulant TF-positive EVs in pediatric embryonal tumor cells. These could regulate the inflammatory state caused by the tumor and serve as a therapeutic mimic^[^27^]^. In another recent study, miR-24-1-5p enhanced angiogenesis in thrombin-exposed adults^[^28^]^. In various other studies, miR-126, miR-16, and miR-155 upregulate the VEGF signaling pathways, promoting angiogenesis in tumor cells, thus being a valuable therapeutic and diagnostic tool^[^29–31^]^. Long noncoding RNAs (lncRNAs) such as MALAT1 and H19, through the process of transcription and the sponging mechanism, increase thromboinflammation and angiogenesis in cancer patients^[^32,33^]^. circRNAs, on the other hand, modulate VEGF and MMPs by competing with miRNAs^[^34,35^]^. miR-155 modulates the endothelial VEGF and inflammatory cytokines, regulating the microenvironment of tumor cells, causing immune dysregulation^[^31^]^. Antagomirs that target miR-24, miR-155, and circRNAs might help in the reduction of the vascularity of cancer cells^[^28,31,34,35^]^. Circulating levels of miR-16, miR-126, H19, and circRNAs could be used as potential biomarkers of prothrombotic state and hyperinflammatory state caused by cancer cells^[^29,30,33–35^]^. A table summarizing the above findings and elaborating further on the topic is illustrated below in Table 1.

**Table 1:** Noncoding RNAs in cancer.

RNA	RNA subtype	Pathophysiology	Function/utility	Tumor	Reference
miRNA	miR-520g	Decreased TF, decreased emission of TF as cargo of EV	Decreased procoagulation/therapeutics	Paediatric embryonal brain tumor	^[^27^]^
miRNA	miR-24-1	Downregulates tumor suppressor genes and upregulates proto-oncogenes	Angiogenesis/targeted therapeutics	Leukemia, Lymphoma, Retinoblastoma	^[^28^]^
	miR-24-2				
miRNA	miR-16	Binds VEGF	Angiogenesis and inflammation/diagnostic marker	Solid tumors	^[^29^]^
miRNA	miR-126	Targets VEGF-A-mRNA	Angiogenesis/diagnosis	Breast cancer	^[^30^]^
miRNA	miR-155	Endothelial and tumor milieu remodeling	Angiogenesis/diagnostics and therapeutics	Colorectal Cancer	^[^31^]^
lncRNA	MALAT1	Inflammation and proliferation	Angiogenesis and thromboinflammation/biomarker and therapeutics	Prostate, lung, breast cancer	^[^32^]^
lncRNA	H19	Sponges miR-342 → proliferation and angiogenesis	Angiogenesis/therapeutics	Glioma	^[^33^]^
circRNA	circ-DICER1	Sponges miR-103a-3p → cell migration and tube formation	Angiogenesis/therapeutics	Glioma	^[^34^]^
circRNA	circ-ATXN1	Sponges miR-526b-3p → ↑MMP2/VEGFA	Angiogenesis/therapeutics	Glioma	^[^35^]^

## EVs as mediators and biomarkers

### Types of EVs: microvesicles, exosomes

EVs are now recognized as key messengers in cell-to-cell communication through the delivery of molecular cargo^[^36^]^. The two main classes of EVs are microvesicles (MVs) and exosomes^[^37^]^. MVs range in size from 100 nm to 1 μm and are secreted directly from the plasma membrane, carrying cytoplasmic content. Exosomes, on the other hand, measure between 40 and 120 nm and are released following the fusion of multivesicular bodies with the plasma membrane^[^38^]^.

Both MVs and exosomes contain various types of proteins, lipids, and nucleic acids, including cell surface receptors, cytosolic signaling proteins, transcription factors, metabolic enzymes, extracellular matrix proteins, RNA-binding proteins, RNA transcripts, miRNAs, and genomic DNA fragments. In this context, EV-mediated signaling between cancer cells may promote cell growth and survival, shape the TME, and increase disease aggressiveness and dissemination. This foundational understanding of EV types sets the stage for exploring their roles in cancer-related blood clotting and new blood vessel formation^[^37^]^.

### EV cargo (RNAs, TF, VEGF) in coagulation and angiogenesis

Available evidence shows that EVs play a critical role in both physiological and pathological pathways of coagulation and angiogenesis^[^39^]^. TF is responsible for initiating coagulation and is not expressed within the blood under physiological conditions. However, in cancer patients, there can be high concentrations of MVs exposing TF, which predispose not only to thrombosis but also to disease progression. While MVs are well-known to contribute to coagulation, the involvement of exosomes is still being explored and may depend on cancer type^[^40^]^.

Interestingly, many of the signals and pathways involved in clotting also contribute to the formation of new blood vessels (angiogenesis), particularly under the influence of cancer-driven inflammation. In this sense, some studies have already shown that MVs produced by ECs and platelets can stimulate angiogenesis through processes mediated by VEGF and other growth factors, such as FGF and PDGF, a process that can promote tumor angiogenesis and metastasis. MVs can also act as inhibitors of angiogenesis through pathways that stimulate the production of reactive oxygen species and inhibit nitric oxide production. It has also been demonstrated that tumor cells can release exosomes that promote angiogenesis. For example, glioblastoma cells have been shown to release exosomes carrying mRNAs and miRNAs that promote blood vessel formation and tumor aggressiveness^[^40,41^]^.

### Pediatric cancer EV studies

Recent studies are starting to uncover how EVs help pediatric tumors grow and spread by reshaping the immune and vascular environment around them. For instance, EVs play a key role in altering the bone marrow microenvironment, enabling the formation of a pre-metastatic niche in the context of neuroblastomas and acute lymphoid and myeloid leukemias^[^42^]^. Some exosomal miRNAs, such as miR-21 and miR-155, are being investigated as potential indicators of how aggressive a tumor might be – but more research is needed in children^[^43^]^. These include solid tumors such as osteosarcomas, rhabdomyosarcomas, Wilms tumor, retinoblastoma, hepatoblastomas, sarcomas (such as Ewing sarcoma, rhabdoid tumors, and non-rhabdomyosarcoma soft tissue sarcomas), neuroblastoma, and other brain tumors, as well as hematologic tumors such as lymphomas and leukemias^[^43–48^]^.

### Potential as liquid biopsy tools

Liquid biopsy is a less invasive method that analyzes blood or other body fluids to find tumor-related material like DNA, RNA, and EVs, thereby informing cancer diagnosis, treatment, and prognosis^[^49^]^. EV-based liquid biopsies have shown promise in detecting early tumor stages, monitoring minimal or residual disease, and tracking post-therapy outcomes in various pediatric cancers^[^42^]^. Some examples are neuroblastoma, Ewing sarcoma, rhabdomyosarcoma, medulloblastoma, and osteosarcoma^[^42,50^]^. Therefore, using EVs as part of a liquid biopsy may help doctors detect cancer earlier, monitor how well treatment is working, and catch signs of relapse in children.

## Epigenetic modifiers of hemostasis and angiogenesis

Epigenetics are heritable, yet reversible modifications in gene expression that play a pivotal role in normal development and disease progression. They are now known to influence key processes in cancer development, including key genes involved in processes such as coagulation and angiogenesis, especially in the setting of pediatric tumors. Their distinct molecular characteristics may amplify the influence of epigenetic alterations on tumor behavior and progression. Dysregulation of the genes in crucial biological pathways in pediatric cancers can contribute to an abnormal TME by promoting inflammation, thrombosis, and pathological neovascularization, all of which support tumor growth and progression^[^51,52^]^.

### DNA methylation and hemostatic gene dysregulation

DNA methylation is an important epigenetic mechanism regulating gene expression. Any disruption in the process of methylation, especially of the CpG islands on the gene promoter regions, can either silence or enhance expression^[^51^]^. In pediatric cancers, normal methylation patterns are affected, leading to dysregulation of several genes involved in coagulation pathways.

Epigenetic alterations that lead to hypomethylation of the promoter regions of F3 (TF) and SERPINE1 (PAI-1) result in overexpression, contributing to an imbalance in the procoagulant and fibrinolytic processes that promote tumor growth and vascular dysfunction^[^53–56^]^. The F3 (TF) gene, a key initiator factor of the extrinsic coagulation pathway, is frequently overexpressed in pediatric tumors, such as leukemia and neuroblastoma, with evidence linking this to decreased methylation in its promoter and early coding regions^[^53–55^]^. Similarly, SERPINE1, which regulates fibrinolysis, shows elevated expression in certain pediatric leukemias due to promoter hypomethylation, contributing to a prothrombotic, tumor-promoting environment^[^56^]^.

Other coagulation-related genes are also subject to methylation-associated epigenetic modification, though they are less extensively studied. TFPI (TF pathway inhibitor), an important regulator in anticoagulation and extracellular matrix production, is hypermethylated in certain pediatric tumors^[^57^]^. As a result, the gene’s promoter activity is suppressed, and thereby reduces the inhibitory effects on coagulation and matrix remodeling. Decrease in TFPI expression can enhance tumor cell adhesion and invasiveness, facilitating metastasis and tissue infiltration^[^57^]^. PLAU, a gene encoding urokinase-type plasminogen activator, is often hypomethylated in pediatric malignancies, resulting in its upregulation^[^58^]^. Overexpression of this gene promotes extracellular matrix degradation and increased cell motility. These disruptions are closely linked to metastatic spread and may contribute to immune invasion by increasing immune cell infiltration and antigen presentation^[^58^]^.

The changes, driven by DNA methylation, can affect several genes involved in the coagulation cascade, highlighting the role of epigenetic mechanisms in shaping the tumor environment in pediatric cancers by promoting inflammation and abnormal blood clotting.

### Histone modifications in tumor angiogenesis

In addition to methylation-associated tumorigenesis, histone modifications, such as acetylation and methylation, also play critical roles in controlling the expression of genes involved in angiogenesis. Modifications to histone proteins help shape chromatin structure and control how accessible the DNA is for transcription. These mechanisms have been associated with the altered expression of proangiogenic factors^[^59,60^]^.

Angiogenesis is a critical process in tumor development, mainly driven by HIF-1α, a key regulator of neovascularization in hypoxic conditions. Histone deacetylase (HDAC) enzymes modify histones to stabilize HIF-1α, thereby extending its activity in low-oxygen environments like that of a tumor^[^61^]^. This further upregulates VEGF expression, an important proangiogenic factor, and promotes tumor angiogenesis^[^61,62^]^.

Among the HDAC enzyme family, HDAC-5 specifically regulates angiogenesis by deacetylating HIF-1α chaperones, diminishing the inhibitory control of FGF-2, another proangiogenic factor^[^63^]^. By enhancing HIF-1α stability and activity, HDAC-5 upregulates the gene expression that drives angiogenesis. In contrast, a histone methyltransferase enzyme, EZH2-mediated H3K27 trimethylation, on a histone protein, plays a central role in silencing antiangiogenic genes in pediatric cancers. This epigenetic repression tips the balance in favor of vascular proliferation, particularly in T cell acute lymphocytic leukemia (T-ALL)^[^64^]^.

Beyond individual gene regulation, epigenetic complexes influence the TME at a broader level. The microenvironment exhibits distinct epigenetic profiles that can influence local hemostasis and angiogenic activity. MYBL1 activates ANGPT2 by recruiting a histone methylation complex (composed of PRMT5, MEP50, and WDR5) to the ANGPT2 promoter. This boosts its expression and destabilizes the vascular integrity to increase the metastatic potential of the tumors^[^65^]^. These changes help remodel the TME, enhancing vascular growth, promoting immune evasion, and supporting the aggressive behavior of some pediatric cancers.

### Epigenetic drugs: current/future therapeutic implications

Integration of epigenetic therapies into pediatric cancer treatment is an emerging area. Understanding how epigenetic changes influence hemostasis and angiogenesis opens opportunities for targeted therapies. Drugs such as DNMT inhibitors (e.g., decitabine), HDAC inhibitors (e.g., panobinostat), and EZH2 inhibitors (e.g., tazemetostat) can suppress prothrombotic and proangiogenic gene expression by targeting epigenetic modifiers, thereby normalizing the tumor vasculature and reducing the risk of metastasis^[^66,67^]^.

Monotherapies have limited durability because of overlapping silencing mechanisms and other compensatory epigenetic adaptations that allow tumors to evade sustained response. This prompted interest in combined regimens with chemotherapy. These agents may help reprogram the tumor’s epigenome rather than directly killing the cells. Traditional response criteria may not fully capture their benefit, highlighting the need for epigenetic biomarkers and functional assays. Future therapies may benefit from the development of biomarker-driven stratification and isoform-specific epigenetic inhibitors tailored to pediatric biology^[^66^]^.

Successful translation of these strategies will depend on careful clinical trial designs that balance efficacy with safety in the pediatric population. Given the broad roles of epigenetic regulators in growth and tissue differentiation, long-term follow-up will be essential to assess developmental outcomes. Advances in our understanding of how epigenetics drives vascular and coagulation pathways in pediatric cancers are opening new avenues for precision therapies. These approaches aim not only to slow tumor growth but also to correct the thromboinflammatory environment fueling it.

## Clinical implications and future directions

New discoveries in ncRNAs, epigenetic changes, and EVs in pediatric cancers are opening new paths for improving the management of pediatric cancers, especially in assessing and addressing thromboinflammatory and angiogenic processes^[^47,68–70^]^. These molecular elements impact coagulation, vessel integrity, and tumor vascularization, and many have shown potential as biomarkers for diagnosis and prognosis^[^71–73^]^.

ncRNAs, including microRNAs and lncRNAs, are increasingly recognized as valuable diagnostic and prognostic biomarkers in pediatric cancers, given their roles in regulating tumor progression, metastasis, and therapeutic response^[^69,74,75^]^. Their stability in body fluids and tissue-specific expression patterns make them especially useful for noninvasive monitoring and individualized risk assessment^[^69^]^. Several ncRNAs have been associated with specific pediatric tumor types, highlighting their potential as signals of their diagnostic or prognostic value^[^69,74–76^]^.

For example, changes in the miR-17 ~92 cluster are linked to tumor-promoting activity and angiogenesis by inhibiting tumor suppressor pathways like PTEN and TGFβ in neuroblastoma and osteosarcoma^[^77–80^]^. In medulloblastoma, some lncRNA have distinct expression patterns associated with different patient outcomes^[^69,81^]^. In childhood T-ALL, these RNAs influence how cancer cells multiply and respond to drugs, making them valuable tools for diagnosis and disease tracking^[^74^]^.

Personalized risk stratification in pediatric cancer is evolving beyond traditional clinical scoring systems to incorporate molecular biomarkers that reflect tumor biology and vascular status. Traditional risk identification models often do not recognize the molecular complexity of pediatric tumors, especially in predicting thrombosis or relapse^[^82–84^]^.

Molecular markers such as epigenetically regulated genes and EV-associated proteins, including TF and angiopoietin-2 (ANGPT2), may offer earlier and more accurate risk identification^[^53,85,86^]^. TF initiates the coagulation cascade, and ANGPT2 promotes vessel destabilization, both of which are located in EVs^[^73,87^]^. These factors are regulated epigenetically or through ncRNAs and can serve as early indicators of thromboinflammatory risk or help evaluate responses to antiangiogenic treatments^[^53,85,86^]^.

Multi-omic panels can integrate epigenetic, transcriptomic, and proteomic data to offer a comprehensive approach to risk assessment and treatment monitoring in pediatric cancer^[^88,89^]^. Techniques like DNA Methylation profiling, RNA sequencing, and EV proteomics analyze molecular features to capture complex tumor biology^[^89–92^]^. Combined with functional drug profiling, it may be possible to enhance the precision of diagnosis, improve risk assessment, and tailor treatment strategies^[^89,93^]^.

The minimally invasive multi-omics panel makes it a convenient tool for pediatric patients as it only requires a small amount of bodily fluids rather than a more invasive tissue biopsy^[^7,94^]^. Analyzing biomarkers from accessible biological samples like blood and other bodily fluids enhances clinicians’ ability to track changes in tumor behavior and adjust therapeutic responses accordingly^[^7,94^]^. Furthermore, it offers critical molecular insights, assists in real-time treatment response and prognosis monitoring, and enables personalized clinical care^[^89,93^]^.

Advancing these molecular insights into practical clinical tools will require coordinated, multidisciplinary pediatric research efforts. These efforts will focus on confirming the predictive value of candidate biomarkers and shaping personalized approaches to control tumor development and thromboinflammatory complications in children with cancer. The essential next step is to test the potential of these biomarkers in large-scale pediatric studies, develop standardized protocols for clinical application, and incorporate them into real-time patient care.

## Conclusion

Pediatric cancers exhibit distinct molecular and developmental features compared to adult malignancies, with thromboinflammation and angiogenesis emerging as critical components of tumor progression. The interplay between coagulation, inflammation, and vascular remodeling not only sustains tumor growth but also contributes to metastasis and treatment-related complications. Despite the high thrombotic risk observed in children with cancer, much of the mechanistic understanding remains extrapolated from adult studies, underscoring the need for pediatric-specific research.

Recent findings have identified ncRNAs, EVs, and epigenetic modifications as key regulators of these interconnected pathways. MicroRNAs and lncRNAs modulate genes involved in coagulation and angiogenesis, while EVs facilitate communication within the TME through the delivery of TF, VEGF, and regulatory RNAs. Epigenetic changes, including DNA methylation and histone modifications, further influence the expression of prothrombotic and proangiogenic genes.

Together, these molecular elements offer valuable opportunities for biomarker development, noninvasive monitoring, and targeted therapies. The integration of multi-omic profiling with conventional diagnostic tools has the potential to enhance risk stratification and guide personalized treatment strategies in pediatric oncology. In particular, liquid biopsy platforms based on EVs and RNA signatures could enable earlier detection of thromboinflammatory complications and real-time tracking of treatment response.

Moving forward, translating these insights into clinical practice will require well-designed pediatric trials and standardized validation of candidate biomarkers. A deeper understanding of the vascular and inflammatory dimensions of pediatric tumors holds promise for improving both survival outcomes and long-term quality of life.

Lastly, this narrative review adheres to the Transparency in the Reporting of Artificial Intelligence in Research (TITAN) guideline^[^95^]^. During the preparation of this manuscript, generative AI tools (DeepSeek, DeepSeek Inc, June 2025 version: temperature parameter = 0.7) were used only as auxiliary aids to assist in formatting, standardization, organization of background information, and language consistency checks. Furthermore, AI-assisted text was thoroughly reviewed, edited, and refined by the authors. The conceptualization, literature, interpretation, synthesis of ideas, and manuscript writing were performed entirely by the authors, ensuring the academic rigor, accuracy, and originality of the review.

## Data Availability

All data discussed in this narrative review are derived from previously published studies and publicly available literature. No new datasets were generated or analyzed for this study.

## Research registration unique identification number (UIN)

Not applicable. This manuscript is a narrative review and does not involve primary clinical research or interventional studies requiring prospective registration.
